# Optimization of Growth Conditions to Produce Sustainable Polyhydroxyalkanoate Bioplastic by *Pseudomonas aeruginosa* EO1

**DOI:** 10.3389/fmicb.2021.711588

**Published:** 2021-10-15

**Authors:** Richa Prasad Mahato, Saurabh Kumar, Padma Singh

**Affiliations:** ^1^Department of Microbiology, Kanya Gurukul Campus, Gurukul Kangri University, Haridwar, India; ^2^Molecular Bioprospection Department, CSIR-Central Institute of Medicinal and Aromatic Plants, Lucknow, India

**Keywords:** *Pseudomonas aeruginosa*, groundnut oil, biodegradable thermoplastic, FTIR, GC-MS

## Abstract

Polyhydroxyalkanoates (PHAs) are intracellularly synthesized by bacteria as carbonosomes that exhibit biodegradable thermoplastics and elastomeric properties. The use of cheaper edible oils as a source of carbon assists in the reduction of the production cost of such biopolyesters. In this work, different edible oils, such as groundnut oil (GNO), mustard oil, sesame oil, and soybean oil (SBO) were used to check their effect on PHA production from *Pseudomonas aeruginosa* EO1 (MK049902). *Pseudomonas aeruginosa* EO1 was used in a two-stage production system. In the first stage, bacterial growth was favored and, in the second, PHA was synthesized. GNO was found as the best carbon source for PHA production. The use of 2% (v/v) GNO, rich in saturated fatty acids, allowed PHA content of 58.41% and dry cell weight (DCW) of 10.5g/L at pH7 and temperature 35°C for 72h. Groundnut has a high potential for oil production and for the diversification of co-products with some potential of value aggregation. Such a perennial and sustainable species will almost certainly meet the criteria for becoming a significant commercial oilseed crop. Fourier transform infrared spectroscopy (FTIR) spectra showed strong characteristic bands at 1,282, 1,725, 2,935, 2,999, and 3,137cm^−1^ for the PHA polymer. Gas chromatography-mass spectrometry (GC-MS) detects the presence of PHA copolymers.

## Introduction

Products that arise from synthetic plastic generate serious difficulties in solid waste disposal when dumped into the environment. However, it is difficult to decline the utilization of plastic products because of their versatile and widespread applications, but it is possible to explore the substitute of conventional petrochemical-based plastics with biodegradable biomaterials (Mohapatra et al., 2015). Nowadays, Biopolymers have focused much public and industrial attention due to great discussions searching for better waste-management plans (Doi, 1990). PHA are good members of biodegradable polyesters for the production of eco-friendly, biodegradable plastics. It is synthesized inside a diversity of microorganisms as a carbon and energy reserve, and its synthesis depends on environmental stress conditions e.g., a deficiency of nitrogen, phosphate, or oxygen (Lee, 1996a). The utilization of biodegradable polymers permits composting as a supplementary way for waste management. Polyhydroxyalkanoate (PHA) is an organic polyester with commercial properties, such as being eco-friendly, biodegradable, thermoplastic, and a biomaterial (Ramsay et al., 1990). Moreover, the utilization of these polymers connects with various other applications in medicine, agriculture, and industry. At least 75 genera of distinct Gram-positive and Gram-negative bacteria have the ability to produce and accumulate intracellular natural polyesters, PHAs, as energy reserve material (Sangkharak and Prasertsan, 2008). To maintain their existence in a changing environment, many bacteria adopted a strategy of intracellular accumulation of storage polymers. PHA depositing capability is an example of this feature and generally indicates a short-termed abundance of carbon sources in contrast to other nutrients such as nitrogen and phosphorous (Steinbüchel et al., 1992). The prime hindrance for the commercialization and production schemes of PHAs is mainly the cost of the carbon substrate used. Bacteria could distribute into two groups according to their PHA accumulation strategies. The first group includes those bacteria that produce PHA when subjected to nutrient limitations, for instance, *Ralstonia eutropha* or *Pseudomonas oleovorans*. The second group includes the bacteria that do not need nutritional limitations for PHA synthesis during bacterial cell growth, for instance, *Alcaligenes latus*, *A. vinelandii*, *Pseudomonas putida*, c47T2, and r-*E. coli* (Lee, 1996a).

Plant oils, such as soybean, palm, and corn oil are suitable carbon substrates for PHAs synthesis because of their low cost compared to most sugars (Ciesielski et al., 2015). The theoretical yield of PHA production from fatty acids is 0.65g/g, while the PHA yield from glucose ranges between 0.30 and 0.40g/g (Chanprateep, 2010). Carbohydrates, pure alkanes, and fatty acids are the common carbon substrates utilized in PHA production (Jiang et al., 2008). An average calculation for each tonne of polymer produced equals about three tons of glucose used (Collins, 1987). There could be various factors responsible for the higher PHA production by vegetable oils as (a) high carbon amount per unit mass in contrast to sugars (Chee et al., 2010) (b) the higher catabolism rate of lipid substrates (beta-oxidation at lipids; Muhr et al., 2013a,b); and (c) and the easy metabolization of saturated and unsaturated fats by microorganisms to produce PHAs (Koller et al., 2012).

In the discussion of PHAs synthesis, *Pseudomonas* sp. is one of the most studied bacteria (Jiang et al., 2008). Additionally, *Pseudomonas* shows a predominant and high growth rate even at a lower cost and, various substrates have been appreciated as good PHA producers. *Pseudomonas aeruginosa* usually produces medium chain length (mcl) PHAs (Soberon-Chavez et al., 2005a). In this paper, we report: (1) The taxonomic classification of the *P. aeruginosa* strain EO1 (MK049902); (2) The PHA accumulation ability of *P. aeruginosa* strain EO1 utilizing edible oils; (3) and also describe the influence of nutritional conditions on its growth; and (4) characterization of produced PHA.

## Materials and Methods

### Bacteria and Culture Medium Used

The strain EO1 used in this study was isolated from edible-oil-contaminated soil from the Haridwar district, India. The Tributyrine hydrolysis, Nile blue, and Sudan Black staining method was used for direct screening of bacteria that accumulate PHA. EO1 was characterized by colony characteristics, gram staining, and biochemical test as *Pseudomonas* sp. (Singh and Mahato, 2019).

The following were the components used in Mineral salt medium (MSM) for PHA production in g/L: 20ml GNO, 1.5g KH_2_PO_4_, 3.57g Na_2_HPO_4_, 0.2g MgSO_4_.7H_2_O, 1g (NH_4_)SO_4_, and 1ml trace element solution prepared following the previous procedure given by (Grothe et al., 1999).

### Molecular Identification of Isolated Bacteria

16S rRNA sequencing was the molecular method used to identify PHA-producing bacteria carried out at Macrogen, Seoul, Korea. For this, genomic DNA was isolated using a Montage PCR product clean-up Kit. The 16S rRNA gene was then amplified by universal eubacterial primers 27F and 1492R. 27F 5'AGAGTTTGATCMTGGCTCAG3' and 1492R 5'TACGGYTACCTTGTTACGACTT3' primers proceeded the Forward and reverse DNA sequencing reaction of PCR amplicon. Thermal cycling consists of three steps: 95°C for 3min, followed by 30cycles of 95°C for 30s, 56°C for 30s, 72°C for 45s, and a final step of 72°C for 10min. The PCR products had proceeded for sequencing. By following the manufacturer’s instructions, the amplified DNA fragments were gel-purified utilizing QIA quickTM Gel Extraction Kit (Qiagen, United States) and sequenced by Macrogen Inc. (Seoul, Korea) using an ABI3730 XL Automatic DNA Sequencer (Applied Biosystems, Renton, United States). Finally, neighbor-joining was the method applied for phylogenetic tree construction on MEGA 7.0.20. References sequences with accession numbers were retrieved from GenBank and depicted on the phylogenetic tree (Tamura et al., 2013).

### Characterization of PHA Potent Bacteria by FE-SEM Analysis

The difference between the PHA accumulating bacterial cells that grow in nutrient-deficient medium *viz* (MSM) and non-PHA accumulating bacterial cells that grew in nutrient-rich medium *viz* (NB) was observed by the Field Emission Scanning Electron Microscopy (FE-SEM). Firstly, extracted bacterial cells from NB and MSM were removed by phosphate-buffered saline (PBS), then fixed in 3% glutaraldehyde solution overnight. The fixed bacterial cells were again washed in PBS to withdraw excess glutaraldehyde and sequentially dehydrated in 30, 50, 70, 80, and 100% ethanol. About 5μl of this bacterial cell suspension was sputter-coated with gold and analyzed in FE-SEM (Carl Zeiss Ultra Plus; Pillai et al., 2017).

### Effect of Growth Conditions for PHA Production

Physicochemical parameters were enhanced for PHA production on different pH (6.0, 7.0, 8.0, and 9.0), temperature (25, 30, 35, and 40°C) and, incubation period (24, 48, 72, and 96h) by the bacteria on a production medium supplemented with MSM and containing Soybean oil (SBO), Groundnut oil (GNO), Sesame oil (SO), and Mustard oil (MO) as carbon substrates and ammonium sulfate, ammonium chloride, ammonium nitrate, and yeast extract as nitrogen sources incubated at 150rpm in the shaker (Lathwal et al., 2015). For the optimization process, 1% of bacterial inoculum was used. Experimental design includes three flasks for each optimization parameter. The pH of the medium was regulated by using 1N HCl or NaOH. Then, the correlation of result *via* measuring the bacterial dry cell weight (DCW) and the extracted weight of PHA.

### Production of PHA Under Biphasic Growth Conditions

Production studies of PHA were performed using 250ml Erlenmeyer flasks containing 100ml of mineral medium supplemented with groundnut oil (GNO) as carbon substrates at 2% concentration and ammonium sulfate as nitrogen source then sterilized the medium by autoclaving at 121°C for 20min. The separation of oil molecules was performed using a sonicator then shaking in an incubator so that bacteria were able to utilize it (Tufail et al., 2017). The activated culture of selected strains was inoculated at 4% (v/v) into MSM for PHA synthesis. Fermentation was performed at 35°C under 150rpm for 72h. Then allow the flask for incubation at 37°C. The 5ml of incubated medium was checked for bacterial DCW and PHA (Abid et al., 2016) at regular intervals of 12–96h from both the control and inoculated medium. The growth as indicated by DCW was plotted against time.

### Measurement of Dry Cell Weight

Centrifuged the bacterial culture to measure the DCW at 8,000rpm for 15min and, the pellet was oven-dried at 55°C to obtain constant weight followed to the cooling and then finally recording the weight (Basavaraj et al., 2013). Determination of the bacterial DCW by subtracting the filter paperweight from the filter paperweight plus the cells. Calculation of the bacterial DCW was by using the formula:


DCW = W2−W1

Where *W2* is the final dry weight of filter paper with cell pellet and *W1* is the initial dry weight of filter paper without pellet (Abid et al., 2016).

### Recovery of PHA

The extraction of the polymer for the quantitative screening of PHA was carried out by inoculating 5% pre-culture broth in 100ml of MSM medium for 72h of incubation at 35°C. Then 10ml of MSM culture was centrifuged at 8,000rpm for 15min, resulting in the formation of the pellet, the cell pellet was incubated at 50°C for 1h in sodium hypochlorite (NaOCl). To obtain cell extract, a cell pellet containing (10ml) sodium hypochlorite was centrifuged at 12,000rpm for 30min after incubation at 50°C for 1h.

The NaOCl containing cell pellet was serially washed through distilled water to remove bleach solution, acetone to remove low molecular weight lipid, and absolute ethanol. After this, the washed pellet was dissolved in 10ml of CHCl_3_ and incubated overnight at 50°C and was dried at room temperature and kept at 4°C and weighed (Singh et al., 2013). Calculation of the PHA% was *via* the below formula.


$$\begin{array}{l} \mathrm{PHA}\;\mathrm{production}\;\left(\%\right)\\ =\frac{\mathrm{Dry}\;\mathrm{weight of extracted}\;\mathrm{PHA}\;\left(g/L\right)\;}{\;\mathrm{DCW}\;\left(g/L\right)}\times 100 \end{array}$$

### Determination of PHA Content and Monomer Composition

#### FTIR Spectrophotometer Analysis of PHA

A 1mg extracted sample of PHA was mixed with KBr. The polymer sample was analyzed by the Fourier transform infrared spectroscopy (FTIR) in the range 4,000–450cm^−1^ (Mattson-1000 model; Getachew and Woldesenbet, 2016).

#### GC-MS Analysis of PHA

Polyhydroxyalkanoate content was determined by methanolysis of monomers according to the previously mentioned method by Shamala et al. (2009). The 10mg cells were exposed to methanolysis in 1ml chloroform, 0.85ml methanol, and 0.15ml concentrated H_2_SO_4_ at 100°C for 140min. Homogenization of the samples with deionized water and used the bottom organic phase for GC analysis (Shimadzu GC-MS QP 2010 Plus). Identification of compounds was using the NIST14 library (Ansari and Fatma, 2016).

## Results and Discussion

### Genotypic Characterization

**Figure 1:**
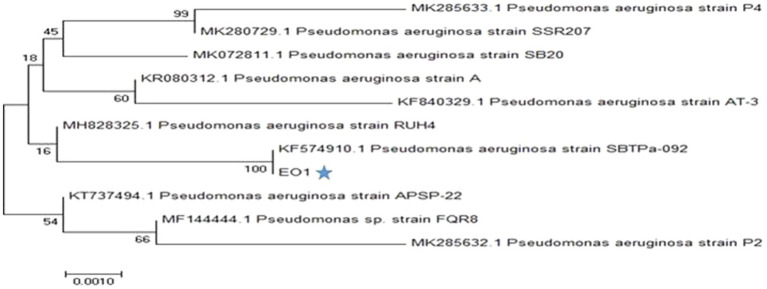
Phylogenetic tree of bacterial strain EO1 Neighbor-joining tree based on 16S rRNA gene sequences showing phylogenetic relationship between strain *Pseudomonas aeruginosa* EO1 and closely related taxa of the genus *P. aeruginosa*.

Bacterial isolate *P. aeruginosa* EO1 was investigated for its taxonomic position. Representation of the species was established upon sequencing of most of the 16S rRNA gene. The closest Pseudomonads strains showed about 98–100% of shared sequence identity with EO1. On analysis of the constructed phylogenetic tree, it was interesting to note that the strain *P. aeruginosa* (EO1) was close to strain KF574910.1 (Figure 1). The analysis of the 16S rRNA gene sequences data for the strain *P. aeruginosa* EO1 shares the same sequence but slightly differs from other *Pseudomonas* species. The partial 16S rRNA sequence of the bacteria to has been submitted to the NCBI gene bank (Accession no. MK049902). Much attempts on research work in this regard by using *P. aeruginosa* for the economical production of PHA, which represents the most common PHA (Aremu et al., 2011; Tripathi et al., 2012; Patel, 2014). The reason could be the predominance of *Pseudomonas* in nature, and its high growth rate even on cheap and distinct substrates. *Pseudomonas* species are characterized by their ability to utilize and degrade a variety of carbon sources due to their wide catabolic versatility and genetic diversity. For these reasons, they are a natural choice regarding techniques of *in situ* and *ex situ* bioremediation (Kahlon, 2016).

### Characterization of PHA Producing Bacterial Cell by FE-SEM

**Figure 2:**
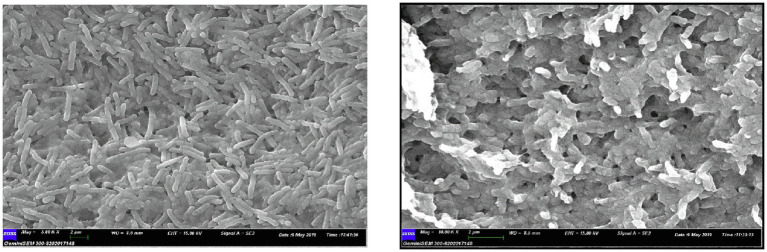
Field Emission Scanning Electron Microscopy (FE-SEM) image analysis of P. aeruginosa EO1 grown on NB **(Left hand)** P. aeruginosa EO1 grown on MSM (**Right hand**).

Field Emission Scanning Electron Microscopy was carried out to detect the PHA accumulating bacterial cells. It indicated that the bacterial cells grown on MSM were large in size and of greater weight while NB-grown cells were of normal shape (Figure 2). In this regard, Lee (1996b) indicated in his work that the bacterial cells grown on MSM were large in size and of greater weight while NB-grown cells were normal in shape. Similar findings were also obtained in previous research by (Bhagowati et al., 2015) that revealed the rod-shaped morphology and a minor rise in the size of isolates after 3days of incubation in Minimal Davis broth under SEM examination. At the optimal growth conditions for *B. cereus* SE-1 using maltose as the only carbon source, cell mass, and PHB accumulation increased with increasing incubation time.

### Influence of Physical Parameters on PHA Production

**Figure 3:**
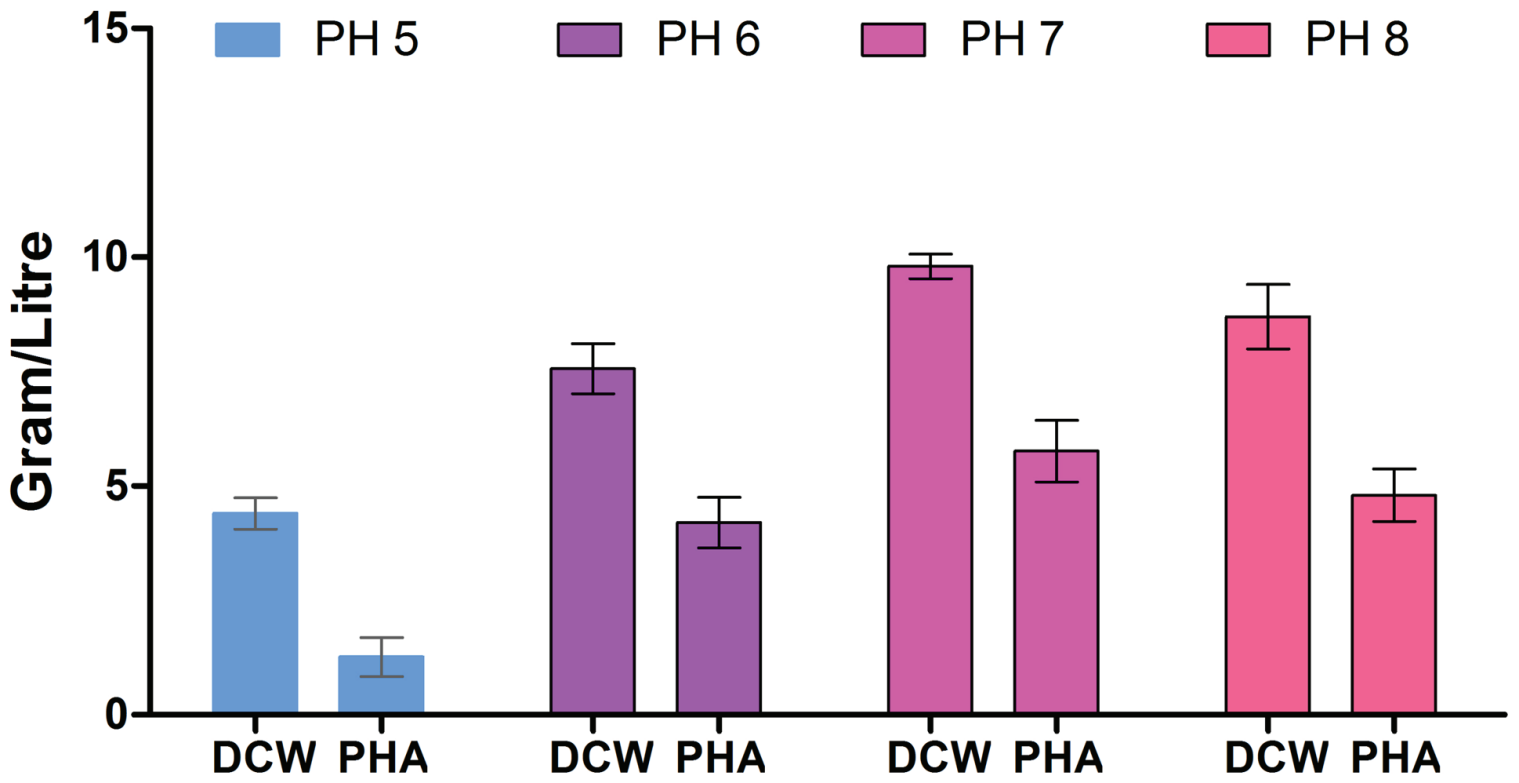
Effect of pH on polyhydroxyalkanoate (PHA) production by *P. aeruginosa* EO1.

The physical growth conditions play a critical role in biomass as well as PHA production. Thus, we optimized the physical growth conditions for PHA production. Figure 3 depicts that bacteria had grown at a vast range of pH (5, 6, 7, and 8) and, the maximum polymer accumulation (58.16%) was at pH 7. This finding is similar to the early reporting for PHA production at pH 7 by *Bacillus subtilis* (Mohapatra et al., 2015). The lower polymer synthesis at extremes of pH (>7 and <7) values is because degradative enzymes break down the polymer so that PHB is utilizing at a rate almost equal to its synthesis rate (Flora et al., 2010).

**Figure 4:**
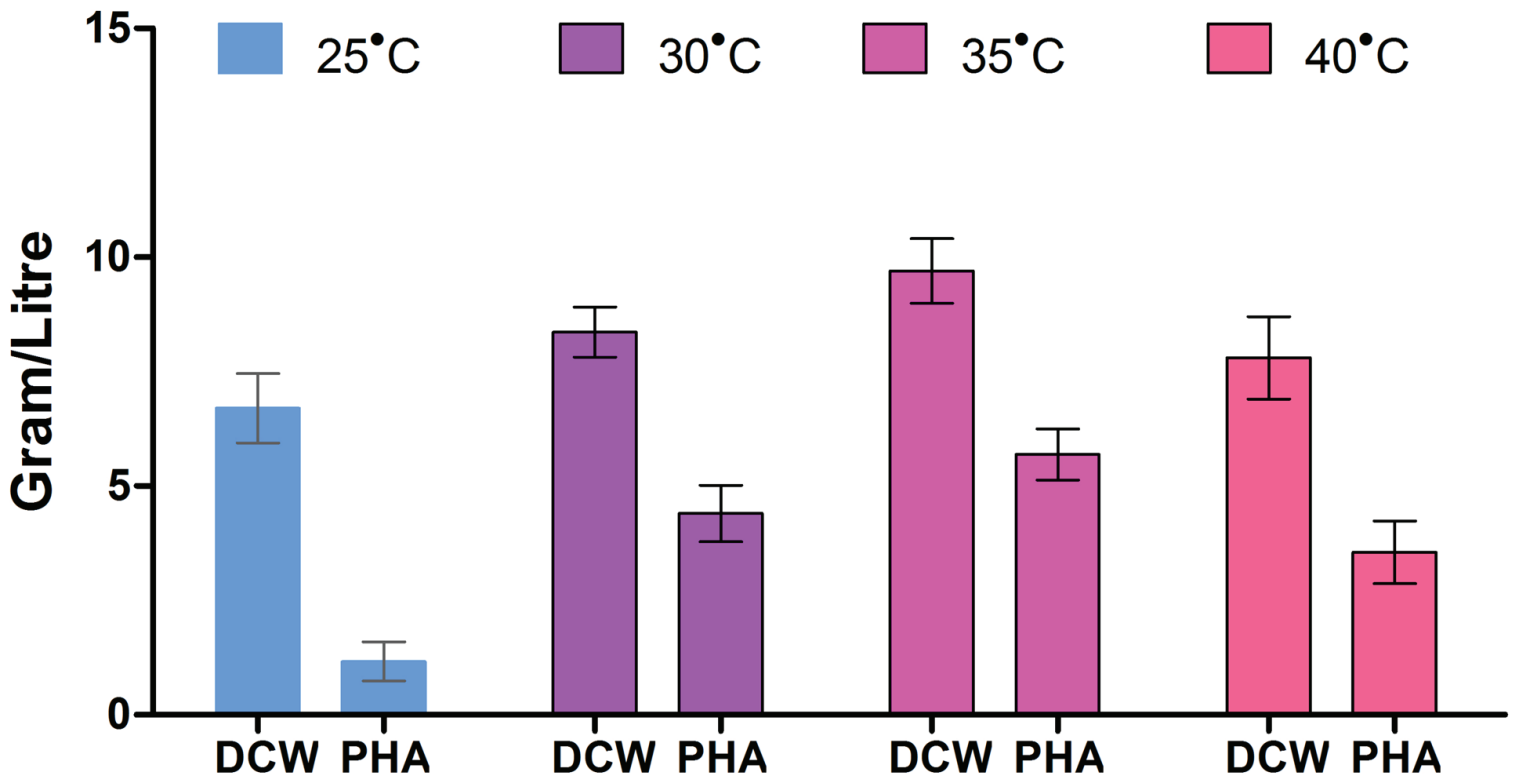
Effect of temperature on PHA production by *P. aeruginosa* EO1.

The influence of varying incubation temperatures (25, 30, 35, and 40°C) was checked, and a maximum of 58.3% of PHA was at the temperature 35±2°C (Figure 4). The PHA accumulation was found to be low at the extremes of the temperature range i.e., (>15°C and<50°C). This response was probably due to the declining activity of enzymes involved in the biosynthesis of PHA at these temperatures. The variation in the temperature could create a change in PHA content, or could be since extreme temperatures minimize the metabolic activity (enzyme activity) of microorganisms, and that ultimately decreases the strength of their PHA production (Noha et al., 2013).

**Figure 5:**
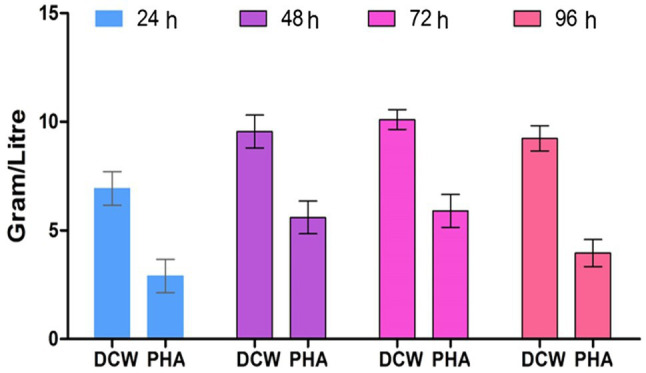
Effect of incubation period on PHA production by *P. aeruginosa* EO1.

The PHA production by strain EO1 was observed by growth in a production medium from 24, 48, 72, and 96h of incubation. PHA accumulation was observed at a maximum of 58.41% at 72h. Enhancement in PHA production occured up to 72h (5.9g/L) of stationary growth phase conditions and, after that, a reduction of 3.96g/L was observed after 72h (Figure 5) due to the intracellular utilization of the PHA as energy reserves and a carbon source, while a hike in metabolites also reduced PHA production (Bhuwal et al., 2013). Regarding the determination of bacterial growth rate and the amount of PHA after 48h of incubation, with each progressive day the bacteria showed various growth rates. The growth relationship on each day was not linear, as the maximum bacterial growth was on the 3rd day of incubation due to the highest density of bacteria.

Our results supported the previous findings as the maximum PHA accumulated at 72h of incubation in *Pseudomonas* sp. (Lathwal et al., 2015). The reason for the reduction in PHA was bacteria using PHA as a carbon source because of nutrient deficiency in the growth medium. Maximum PHA production was observed in isolate AWW at a pH range of 7.0 and temperature 37°C after 48h (Getachew and Woldesenbet, 2016).

**Figure 6:**
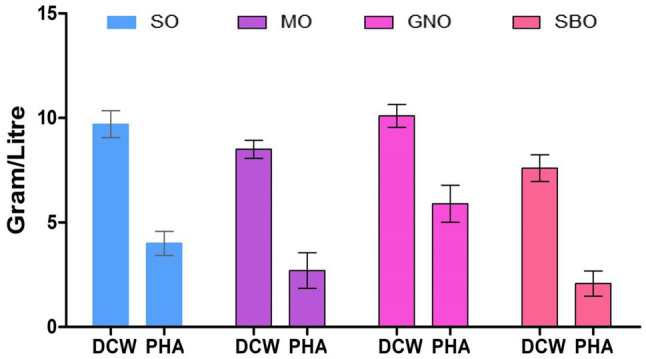
Effect of different carbon sources on PHA production by *P. aeruginosa* EO1.

The influence of four edible oils on PHA production by the bacteria was studied to select the best and most economical one. SBO, GNO, SO, and MO are among the four major vegetable oils produced globally. The GNO was the best carbon source for bacterial growth as it gave (10.1g/L) of PHA (Figure 6). On the other hand, GNO was also the best carbon source for PHAs production (58.41%), followed by SO (40.40%), and MO (31.7%). SBO (27.36%) was the worst carbon source for polymer production. It may be due to the composition of GNO, as it has been reported previously that Peanut oil contains a high concentration of saturated fatty acids (17%) compared to soybean oil (14%) and canola oil (13%; Vassiliou et al., 2009; Pérez-Arauz et al., 2019). The organism exhibits maximum cell growth as it has a selective preference for the carbon source. The reason behind this is a genetic makeup that leads to the metabolism of a specific carbon feed (Ashby and Foglia, 1998).

Ruiz et al. (2019) studied the effect of hydrolyzed waste cooking oil fatty acids (HWCOFA) that showed HWCOFA could be used as the sole substrate for PHA production by *P. putida* KT2440. Glucose was also a suitable substrate for PHA production (Mohapatra et al., 2015).

**Figure 7:**
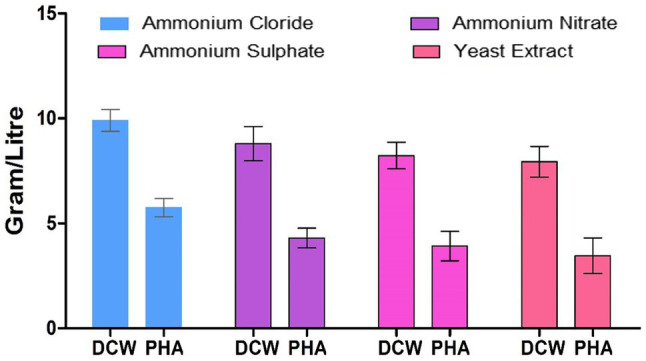
Effect of different nitrogen sources on PHA production by *P. aeruginosa* EO1.

Polyhydroxyalkanoate was produced by the strain EO1 supplemented with four different nitrogen sources (ammonium sulfate, ammonium chloride, ammonium nitrate, and yeast extract), and the identified best carbon source, namely GNO, as illustrated in Figure 7. Out of the four nitrogen sources, ammonium sulfate supported the highest PHA production; giving the maximum PHA accumulation observed by strain EO1 (5.1g/L). This was followed by ammonium chloride (4.3g/L), ammonium nitrate (3.92g/L), and the poorest PHA accumulation was by Yeast extract (3.46g/L).

Previous work reported that out of four nitrogen sources (ammonium chloride, ammonium sulfate, ammonium nitrate, and urea), the highest cell dry mass and PHA level by *Alcaligenes latus* ATCC 29714 (or DSM 1123) was also gained with ammonium sulfate as the nitrogen source (Grothe et al., 1999). Previous work has shown PHA production when utilizing various nitrogen sources (Lathwal et al., 2015). It was noticed that ammonium sulfate increased the yield of PHB produced by strain KS-3. There might be a reason for this in the relatively low nitrogen concentration of ammonium sulfate resulting in an increased C/N ratio, which stimulated an increase PHB synthesis. The least DCW and PHB accumulation were achieved with yeast extract (Basavaraj et al., 2013; Getachew and Woldesenbet, 2016).

### Production and Extraction of Polymer for Estimation of PHA

Among various physicochemical parameters, pH7 and temperature 35°C over the incubation period of 72h was found optimum for maximum PHA production by optimizing different culture conditions. The PHA was extracted from the cell by the hypochlorite and chloroform method as described above. The bacteria exhibited maximum PHA accumulation in MSM (6.1g/L) from 10.5g/L (DCW) under optimized culture conditions and in the presence of the best carbon substrate. Maximum DCW and PHA by isolate EO1 were gained at pH 7 and 72h of incubation in shaken flask culture cultivation. The PHA accumulation was initiated at the initial log phase and continued up to 72h of incubation. The deficiency of nitrogen and the existence of a carbon source enhanced PHA productivity.

Different strains of *Pseudomonas* have been reported previously for PHA production using oily substrates. For e.g., *Pseudomonas* sp. DR2 synthesizes PHAMCL up to 23.53% (w/w) using waste vegetable oil (Song et al., 2008). PHA (50.27% w/w) of the total CDM produced by utilizing soybean oil (Abid et al., 2016). Corn oil serves as a carbon source for bacterial growth and supports cells to synthesize PHA up to 35.63% (Chaudhary et al., 2010). Comparable results were reported by Haba et al. (2007) as *P. aeruginosa* synthesizes PHAs up to 36% (w/w) of the cell dry mass.

### FTIR Analysis of PHA

**Figure 8:**
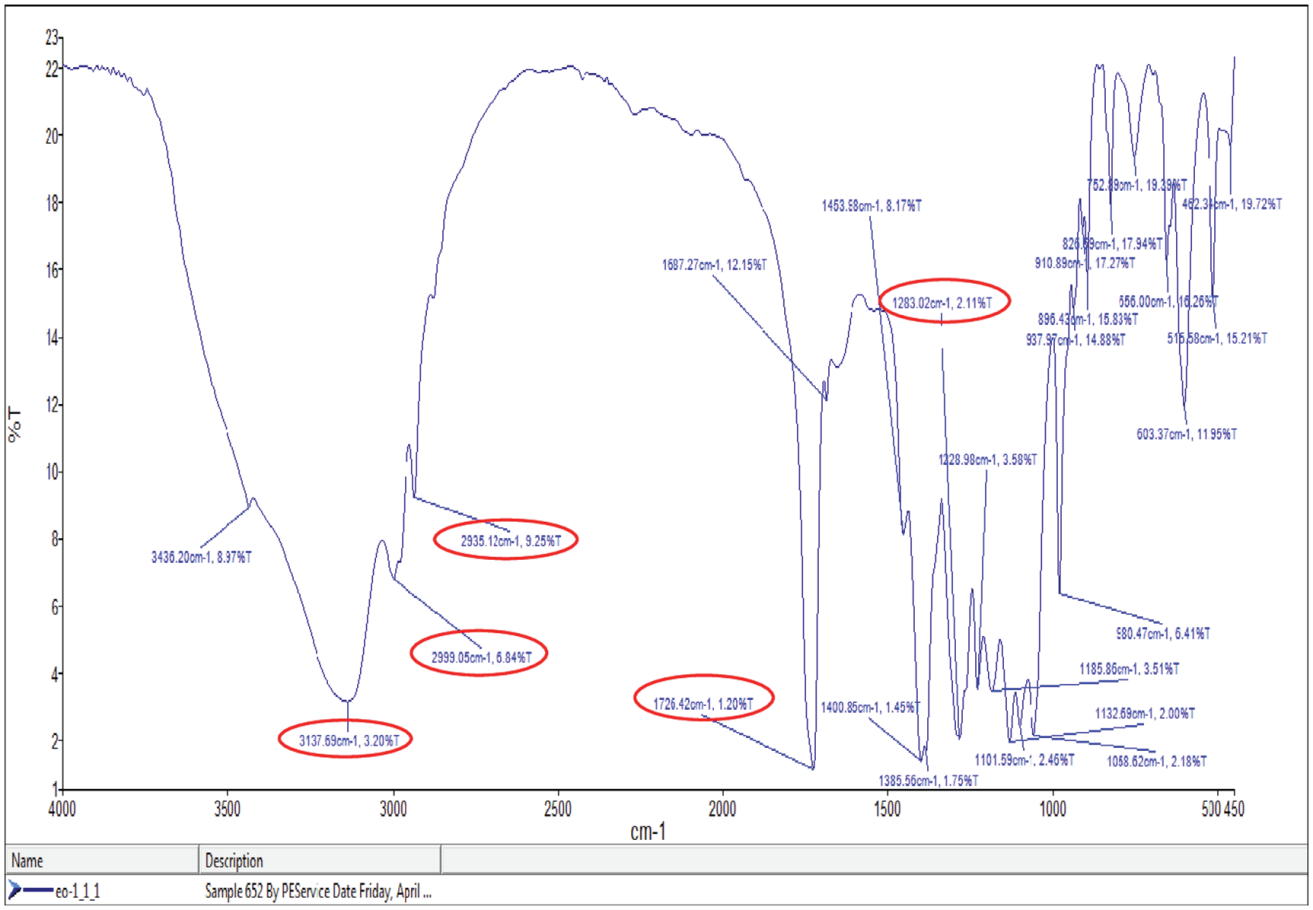
Fourier transform infrared spectroscopy (FTIR) spectra of extracted polymer from *P. aeruginosa* EO1.

Polymer obtained after extraction was applied to record IR spectra in the range between 4,000 and 450cm^−1^ (Figure 8). IR spectra indicated strong absorption bands at 2,935, 2,999, and 3,137cm^−1^ were because of the C-H stretching vibrations of methyl and methylene groups. The absorption bands at 1,283 and 1,726.42cm^−1^ revealed specificity for C-O and ester group (C=O) stretching vibrations. The peaks ranging between 2,848.77–3,008.35cm^−1^ indicated the existence of CH_3_-, -CH_2_- -CH_2_-, and -CH_2_-CH_3_ (Gumel et al., 2014; Abid et al., 2016). Figure 6 showed that the absorption band on 1,726cm^−1^ indicated the PHA marker band appropriated for carbonyl C=O stretches of the ester group detected in the chain of exceedingly arranged crystalline structures (Randriamahefa et al., 2003).

The FTIR result of this study was matched with any prior studied FTIR spectrum of PHA such as Muralidharan and Radha (2014), which had shown two strong absorption peaks of PHB at 1,724.2 and 1,280.3cm^−1^, matching to –C=O and –C-O stretching groups. *Bacillus megaterium* uyuni S29 was used for PHA production and PHA was characterized by IR transmission spectrum. It exhibited main bands at 1,726, 2,960–2,850, 1,390–1,370, and 1,230–1,050cm^−1^ corresponding to the carbonyl group, methyl and methylene groups, the methyl group, and the ester group, respectively. The absorption band 1,385.56cm^−1^ was allocated to the terminal CH_3_ groups and the same group mentioned in the literature at 1,378.83cm^−1^ (Reddy et al., 2008). The peaks near 1,726, 2,935, 2,999, and 1,058.62cm^−1^ confirmed that mcl-PHA copolymer developed because of the variation in the peaks near 2,900cm^−1^ of pure PHB and bacterial synthesized PHAs (Hong et al., 1999; Kim et al., 2007; Khare et al., 2014). These important absorption bands confirmed the structure of mcl-PHA.

### GC-MS Analysis of PHA

**Figure 9:**
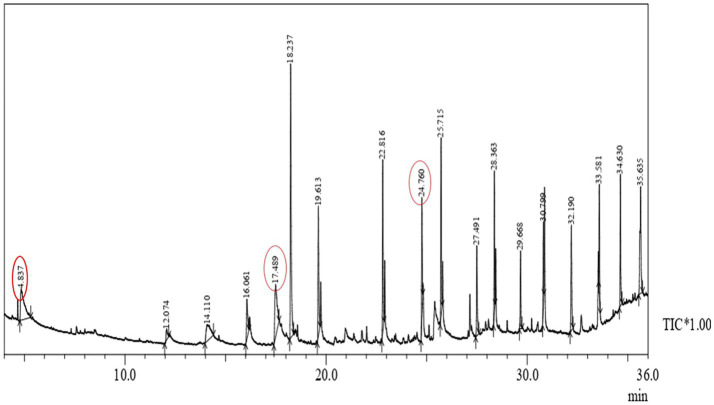
Gas chromatography-mass spectrometry (GC-MS) of extracted polymer from *P. aeruginosa* EO1.

Polyhydroxyalkanoate extracted from *P. aeruginosa* strain EO1 using GNO significantly contained (3HB) Beta-hydroxybutyric acid methyl ester (7.41%), (3HDD) Methyl 3-hydroxytetradecanoate (7.26%), and (3HD) hexadecanoic acid, methyl ester (5.03%). These compounds proved that monomer chains were constructed of a biodegradable polyester family. It is visualized in Figure 9 that the usual molecular fragment of the 3HB ion chromatogram of the PHB developed here. A predominant peak indicated the tetramer of 3HB (Beta-hydroxybutyric acid), Methyl 3-hydroxy-tetra decanoate, hexadecanoic acid, and methyl ester were observed at 4.837, 7.26, and, 5.03min, respectively. The PHA of molecular weight (118kDa) was obtained from *P. aeruginosa* strain EO1 after the GC-MS analysis. About 177kDa, molecular weight of the PHB was recovered by Galego et al., (2000). This drop-down of molecular weight was because of distinct polyhydroxyalkanoic acids, e.g., 3HV and 3HBV up to a low percentage (32%) with the major polymer 3HB. Due to their potential to synthesize mcl-PHA from distinct carbon substrates, Fluorescent pseudomonads are studied well (Huisman et al., 1989; Sun et al., 2007; Goh and Tan, 2012).

## Conclusion

Wide production, commercialization, and thus practical application of PHAs as a biodegradable alternative to conventional plastics is still limited due to high production costs. Plant oils provide an excellent substrate for bacterial growth and PHA production. However, PHA productivity from these substrates needs to be improved in order to progress such processes toward commercial scale and application by optimizing physico-chemical conditions, as these substrates are less costly and more readily scaled. As shown in the present study, all four of the edible oils have tested have potential for PHA production. In the present study, we reported PHA production by *P. aeruginosa* EO1 strain. In the study, the edible oils were used as a source of carbon for *P. aeruginosa* EO1 mediated production of PHA. In addition, we optimized the physicochemical conditions for high yield. Further, the PHA production was confirmed through FTIR spectroscopy and GC-MS analysis. So, we conclude that the *P. aeruginosa* EO1 strains used in this study could successfully utilize cheaply available GNO as the sole carbon source and produced a high level of PHA in flask cultures under optimized culture conditions. Those features will significantly contribute to PHA cost reduction and minimize the requirements for expensive sugar as a carbon substrate.

## Data Availability Statement

The datasets presented in this study can be found in online repositories. The names of the repository/repositories and accession number can be found at: NCBI (accession: MK049902).

## Author Contributions

RM performed the experiments. RM and SK analyzed the data. RM and PS conceived the idea and wrote the manuscript. All authors contributed to the article and approved the submitted version.

## Conflict of Interest

The authors declare that the research was conducted in the absence of any commercial or financial relationships that could be construed as a potential conflict of interest.

## Publisher’s Note

All claims expressed in this article are solely those of the authors and do not necessarily represent those of their affiliated organizations, or those of the publisher, the editors and the reviewers. Any product that may be evaluated in this article, or claim that may be made by its manufacturer, is not guaranteed or endorsed by the publisher.

## Abbreviations

%: Percentage
°C: Degree Celsius
CHCl_3_: Chloroform
DCW: Dry cell weight
EO: Edible oil
FE-SEM: Field emission scanning electron microscopy
FTIR: Fourier transform infrared spectroscopy
GC-MS: Gas chromatography-mass spectrometry
GNO: Groundnut oil
h: Hour
HCl: Hydrochloric acid
mL: Milliliters
min: minute
MO: Mustard oil
MSM: Mineral salt medium
NaOCl: Sodium hypochlorite
NaOH: Sodium hydroxide
NB: Nutrient broth
PHA: Polyhydroxyalkanoate
rpm: Rotation per minute
SO: Sesame oil
SBO: Soybean oil
